# Associations Between Paternal Body Mass Index and Neurodevelopmental–Physical Outcomes in Small-for-Gestational-Age Children

**DOI:** 10.3390/diagnostics15172133

**Published:** 2025-08-24

**Authors:** Yimin Zhang, Shuming Shao, Jiong Qin, Jie Liu, Guoli Liu, Zheng Liu, Xiaorui Zhang

**Affiliations:** 1 Department of Pediatrics, Peking University People’s Hospital, Beijing 100044, China; air685@163.com (Y.Z.); 18801232385@163.com (S.S.); qinjiong@263.net (J.Q.); yy.lovej@163.com (J.L.); guoleeliu@163.com (G.L.); 2 Department of Maternal and Child Health, School of Public Health, Peking University, Beijing 100191, China

**Keywords:** paternal weight, neurodevelopment, social-emotional development, small for gestational age

## Abstract

**Objective:** This study investigated the association between paternal preconception paternal body mass index (BMI) categories and physical/neurodevelopmental outcomes in Chinese small-for-gestational-age (SGA) children. **Methods**: A prospective cohort study enrolled 412 singleton SGA infants born at Peking University People’s Hospital in 2020–2022. Fathers were stratified into underweight, normal-weight, overweight, and obese groups. Follow-up assessments at 24–36 months evaluated growth parameters weight, height, BMI Z-scores and neurodevelopment using the Ages and Stages Questionnaire-3 (ASQ-3) and ASQ: Social–Emotional (ASQ:SE). Multivariable regression was adjusted for paternal covariates. **Results:** In SGA offspring, paternal underweight correlated with lower birth weights vs. normal/obese paternal BMI and the highest severe SGA rates. Prospective monitoring identified elevated BMI Z-scores (ΔZ = +0.40) and 8.7-fold heightened obesity risk in the paternal obesity group versus normal-weight counterparts. Neurodevelopmental evaluations demonstrated gross motor impairments in both underweight (ΔZ = −0.22) and obese paternal subgroups (ΔZ = −0.25) compared with the normal-weight group, with the obesity cohort additionally exhibiting problem-solving deficiencies (ΔZ = −0.19). The paternal obesity group manifested three-fold greater likelihood of social–emotional delays than the normal-weight group. The underweight and obese paternal groups showed 3.46-fold and 2.73-fold higher probabilities of gross motor deficits, respectively, while obesity was linked to 3.27-fold elevated problem-solving impairment risk-all comparisons versus normal paternal BMI. Overweight status showed no significant links to growth or neurodevelopmental outcomes. Normal-weight fathers had lower risks of obesity and neurodevelopmental issues. **Conclusions:** This study revealed U-shaped paternal BMI–neurodevelopment links in SGA offspring. Paternal obesity raised offspring obesity/neurodevelopmental risks, while underweight linked to severe SGA and motor deficits, highlighting paternal weight optimization’s modifiable role.

## 1. Introduction

Small for gestational age (SGA) refers to neonates with birth weights below the 10th percentile for their gestational age and sex [1]. The global prevalence of SGA is approximately 16%, ranging from 7% in developed countries to 27% in low- and middle-income countries, with over 90% of cases occurring in term births [2,3]. In China, the prevalence is 6.4% [4]. Extensive cohort studies have confirmed that SGA not only represents a high-risk population for perinatal mortality but is also associated with adult-onset metabolic syndromes (e.g., obesity and type 2 diabetes), cardiovascular diseases [5,6], and—more importantly—increased susceptibility to physical growth retardation and neurodevelopmental disorders (e.g., cognitive deficits and autism spectrum disorder) [7,8]. While current clinical guidelines [9] emphasize early nutritional interventions and growth acceleration strategies to address postnatal short stature in SGA infants, persistent challenges remain in optimizing neurodevelopmental outcomes [10]. This highlights the need to explore risk regulatory targets from a multidimensional etiological framework.

Traditionally, research on the risk factors for abnormal physical growth and neurodevelopmental outcomes in SGA children has primarily focused on maternal gestational disorders, such as placental insufficiency and genetic factors [11,12], as well as a limited number of studies investigating the correlation with maternal body mass index (BMI) [13,14]. However, recent studies have revealed that paternal weight status, akin to maternal weight status, constitutes a key early-life factor influencing offspring health. Abnormal weight states, including underweight or overweight/obesity, may profoundly influence children’s long-term physical growth and neurodevelopment through mechanisms such as alterations in the intrauterine environment and epigenetic regulation [15,16,17]. Research from Scotland [18] suggested that parental weight status may account for nearly one-third of childhood obesity risk through shared biological and environmental pathways. However, findings diverge when examining early childhood growth patterns: an Indian study [19] reported a detectable link between paternal BMI and infant weight trajectories in children under five years, while Canada’s data [20] from the first two years of life showed no measurable association. Given the differing follow-up periods (up to five years vs. first two years), the comparability of these results is limited, and the certainty of this divergence is reduced. This suggests that paternal influences might emerge at distinct developmental stages or interact with cultural or socioeconomic factors. Neurodevelopmental research amplifies these uncertainties. A large-scale Swedish investigation [21] spanning two decades identified both paternal underweight and obesity as modest but statistically significant contributors to autism spectrum disorder risk. Conversely, a smaller Spanish study [22] analysis found maternal—but not paternal—weight status correlated with neurodevelopmental outcomes. Notably, UK longitudinal data [23] highlighted stronger maternal than paternal BMI effects on cognitive development, suggesting potential differences in biological transmission mechanisms. These findings indicate that existing research has not reached consistent conclusions regarding the relationship between paternal BMI and long-term offspring outcomes. Notably, a significant research gap persists in understanding the paternal nutritional/metabolic determinants of long-term prognosis among SGA children, a vulnerable population requiring special attention. Importantly, paternal BMI—recognized as a modifiable biomarker—may exert transgenerational epigenetic influences on SGA offspring outcomes through mechanisms such as sperm-derived DNA methylation remodeling [24]. Consequently, delineating the paternal BMI–neurodevelopment relationship in SGA children holds dual scientific significance: not only does it advance the etiological understanding of developmental disorders, but more crucially, it establishes an evidence base for implementing paternally targeted preconception-to-perinatal intervention strategies.

This study explores paternal preconception weight status impacts on physical growth and neurodevelopment in Chinese SGA children. The study protocol was approved by the Medical Ethics Committee of the Peking University People’s Hospital (2023PHB353) in accordance with the Declaration of Helsinki. Follow-up assessments of physical, neurocognitive, and socioemotional development aim to identify paternal nutritional influences on long-term outcomes. Results will inform interventions to enhance developmental prognosis.

## 2. Methods

### 2.1. Study Design and Site

This is a prospective cohort study, and the study population comprised neonates delivered in the obstetrics department of Peking University People’s Hospital between 1 January 2020 and 31 December 2022, who were classified as SGA according to Chinese reference standards [25], with birth weights below the sex- and gestational age-specific 10th percentile, while severe SGA constitutes a distinct clinical entity characterized by birth weight measurements under the 3rd percentile of validated population-based growth standards [26]. Between 1 June 2023 and 31 December 2024, we prospectively monitored the physical growth, neurodevelopmental outcomes, and socioemotional behavioral outcomes of singleton SGA children. Singleton SGA cases were stratified into four paternal pre-pregnancy BMI categories according to Chinese criteria [27]: underweight (<18.5 kg/m^2^), normal weight (18.5–23.9 kg/m^2^), overweight (24–27.9 kg/m^2^) and obesity (≥28 kg/m^2^). This study obtained ethical approval from our hospital’s Medical Ethics Committee (Approval No: 2023PHB353), and all patients provided informed consent.

### 2.2. Participants

**Figure 1:**
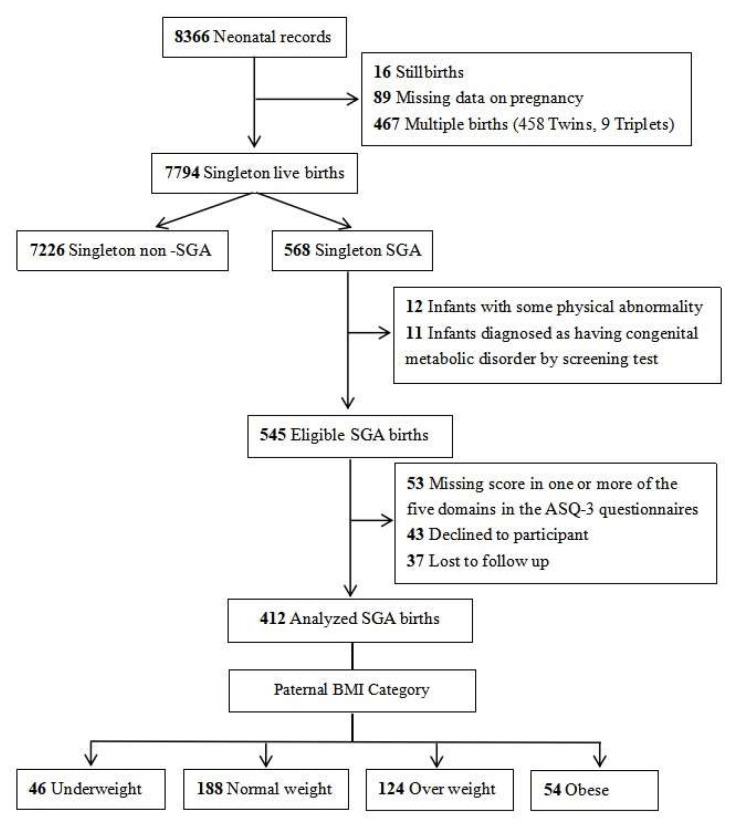
Case selection flowchart.

During the study period, a total of 7794 singleton live-born infants and their parents were registered in our hospital’s obstetrics department. After excluding non-SGA infants, those with congenital malformations and metabolic diseases, cases with incomplete data, and those lost to follow-up, 412 SGA infants and their parents who consented to participate were ultimately enrolled in the study. The detailed participant flowchart is presented in Figure 1.

### 2.3. Perinatal and Parental Data Collection

Perinatal demographic indicators were retrieved from the electronic medical record systems of the obstetrics and neonatology departments. Maternal data were abstracted from prenatal and delivery records, encompassing both baseline characteristics (age, obstetric history, pre-pregnancy height and weight, academic degree, conception method, mode of delivery) and major comorbidities (gestational diabetes mellitus, hypertension, preeclampsia, anemia, hypothyroidism, connective tissue diseases, abnormalities of the umbilical cord, placenta, and amniotic fluid). Gestational weight gain (GWG) quantifies the total maternal weight accumulation from preconception to parturition. According to China-specific GWG criteria, it is divided into three groups: inadequate, adequate, and excessive. For women with different BMIs, the recommended weight gain is 12.5–18.0 kg for underweight women, 11.5–16.0 kg for normal-weight women, 7.0–11.5 kg for overweight women, and 5.0–9.0 kg for obese women [28]. Neonatal records provided sex, gestational age, birth weight, and asphyxia status. Paternal demographic information (age, pre-pregnancy height/weight, and education level) was obtained through outpatient visits and telephone follow-ups conducted for SGA infants.

### 2.4. Sample Size Calculation

The sample size for this study was estimated a priori to ensure adequate power to detect associations between paternal BMI and neurodevelopmental–physical outcomes in SGA children, based on previous research. According to Swedish studies [21], we used these risk differences to calculate the minimum sample size required to detect a clinically meaningful effect, focusing on the comparison between obese and normal-weight fathers as this represents the largest expected difference, with a significance level α of 0.05 and 80% power β = 0.20. Thus, approximately 52 fathers per group were required to detect the risk difference between other groups and normal-weight fathers. However, due to recruitment constraints in the SGA population, the final sample sizes were 54 for obese, 188 for normal-weight, 124 for overweight, and 46 for underweight fathers, with total *n* = 412. Post hoc power analysis using the same parameters indicated that the achieved sample size provided more than 80% power for the obese–normal and underweight–normal comparison, which was deemed acceptable given the study’s exploratory nature and the availability of data on other groups.

### 2.5. Long-Term Physical and Neurodevelopmental Assessment

This prospective cohort study assessed physical and neurodevelopmental outcomes in SGA children with a single follow-up visit conducted at ages 24–36 months (median: 30 months).

Physical development was assessed through weight and height measurements, with BMI calculated from these parameters. To account for age- and sex-related variations, Z-scores standardized anthropometric data across subgroups. Specifically, weight, height, and BMI values were adjusted by subtracting the age- and sex-specific reference population mean and dividing by the corresponding standard deviation. Growth status was evaluated by quantifying deviations from age-matched reference values using these standardized scores [29]. Reference means and standard deviations for weight, height, BMI, and weight-to-height ratio were derived from the nationally representative growth reference curves and anthropometric standards established in the Chinese Child Growth Standards [30]. The diagnostic thresholds for wasting, overweight, and obesity were based on the evidence-based diagnostic framework established in the Eighth Edition of Zhu Futang’s Practice of Pediatrics [31].

Neurodevelopmental evaluations were conducted using the culturally adapted and standardized Chinese versions of two instruments: the Ages and Stages Questionnaire, Third Edition (ASQ-3; 1–65 months) [32], and its complementary module, Ages and Stages Questionnaire: Social–Emotional (ASQ:SE; 3–65 months) [33]. The ASQ-3 operationalizes neurodevelopmental evaluation through five standardized developmental domains: communication, gross motor, fine motor, problem-solving, and personal–social competence. This instrument employs developmentally calibrated questionnaires stratified by chronological age cohorts 1–65 months, enabling domain-specific developmental benchmarking. Domain-specific raw scores underwent norm-referenced standardization using Z-score transformation, calculated through subtraction of age-matched population means, followed by division by corresponding standard deviation values derived from demographically matched reference populations [30]. Scores calculated for each domain were categorized as typical development (above the cutoff value) or referral zone (below two standard deviations). The ASQ:SE serves as a validated screening instrument that systematically assesses developmental trajectories in social–emotional competencies in pediatric populations. This well-validated screening tool is specifically designed to detect developmental deviations from age-normative benchmarks through quantitative thresholds established in standardized reference populations. Clinical interpretation of psychometric data obtained from this screening protocol was rigorously supervised by board-certified pediatric developmental specialists, ensuring adherence to evidence-based diagnostic frameworks for the early identification of social–emotional developmental delays.

### 2.6. Statistical Analysis

All data analyses were performed using the statistical software package SPSS version 25.0. Initial univariate analyses were performed without adjustment for covariates. One-way analysis of variance (ANOVA) was used to compare the four groups for normally distributed continuous variables. Continuous variables that did not conform to a normal distribution were represented by the median and upper and lower quartiles [M (P25, P75)], and multiple independent sample Kruskal–Wallis H tests were used for comparison among the four groups. Categorical variables were represented by the number of cases (*n*) and the rate (%), and the χ^2^-test (with Bonferroni correction or Fisher’s exact test as appropriate) was used for pairwise comparison among groups. To control for potential confounding factors and assess independent associations, multivariate analyses were conducted. Multiple linear regression analysis was performed for continuous outcome variables, adjusting for relevant covariates. For categorical outcomes, multivariate logistic regression adjusting for covariates was employed where appropriate. A statistical significance level of *p* < 0.05 was considered statistically significant.

## 3. Results

A total of 412 fathers with singleton SGA infants who completed the ASQ-3 and ASQ:SE questionnaires were available for analysis (Figure 1). The comparative analysis of parental characteristics and obstetric morbidity patterns across the four paternal pre-pregnancy BMI cohorts demonstrated statistical homogeneity in demographic parameters and complication profiles. Maternal pre-pregnancy BMI, maternal age, and paternal age showed no statistically significant differences among the four groups. Maternal conditions, including gestational hypertension, preeclampsia, diabetes, hypothyroidism, connective tissue disorders, nuchal cord, and placental abnormalities, showed no significant intergroup differences (Table 1).

The general characteristics of newborns stratified by paternal BMI are shown in Table 2. After applying Bonferroni correction for seven comparisons, with the significance level adjusted to α ≈ 0.007, no statistically significant differences were observed for any variables. However, focusing on severe SGA incidence, a trend was observed where the underweight group had higher rates compared to the normal-weight group and overweight group *p* = 0.033, with uncorrected *p* < 0.05. No other significant trends were noted in neonatal characteristics.

Prospective follow-up of SGA children stratified by paternal pre-pregnancy BMI categories revealed distinct developmental patterns during 24–36 months. Anthropometric assessments demonstrated comparable weight-for-age and length-for-age Z-scores across all four paternal BMI groups, with no intergroup differences in the prevalence of wasting or overweight. The obese group had significantly higher BMI Z-scores compared to the normal-weight group (ΔZ−score = 0.40), and the incidence of obesity in SGA children in the obese group was significantly higher than in the normal-weight group (adjusted OR 8.70; 95% CI 1.74–63.62). Neurodevelopmental evaluation using the ASQ-3 questionnaire identified domain-specific disparities: both the paternal underweight group and the obese group showed impaired gross motor development compared to the normal-weight group (ΔZ−score = −0.22 and − 0.25, respectively) and the overweight group (ΔZ−score = −0.23 and −0.26, respectively). Furthermore, the paternal obese group showed impaired problem-solving development compared to the normal-weight group (ΔZ−score = −0.19). No significant intergroup variations emerged in communication, fine motor, or personal–social skill domains. (Table 3).

**Figure 2:**
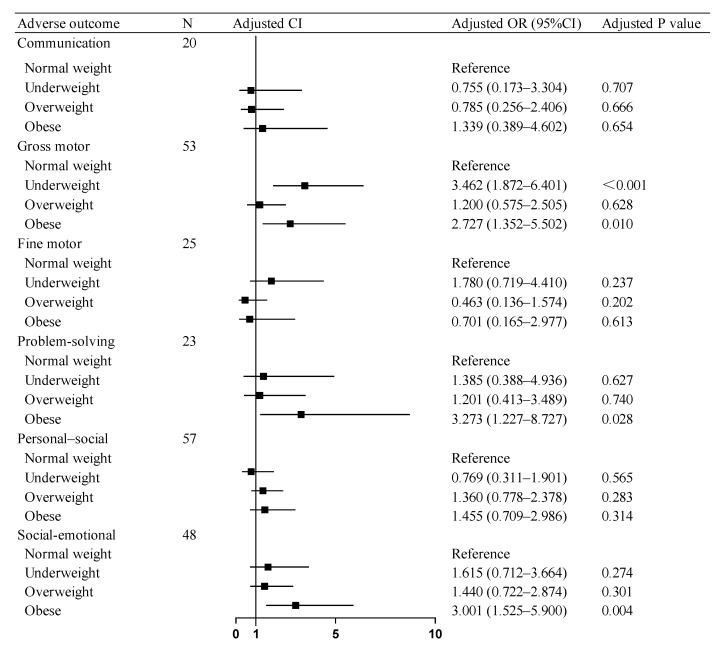
Association of paternal BMI with adverse neurodevelopmental outcomes in SGA children. OR: odds ratio, CI: confidence interval. Abbreviations: BMI, body mass index; SGA, small for gestational age.

The regression models across all domains demonstrated adequate fit in the Hosmer–Lemeshow tests. Multivariable logistic regression analyses revealed that after covariates (including maternal BMI, GWG, diabetes mellitus, and so on) in neurodevelopmental outcomes, SGA children in the paternal obesity group showed elevated risks of adverse consequences compared to the paternal normal-weight group: a 2.727-fold higher risk for gross motor skills and a 3.273-fold increased risk for problem-solving. Concurrently, the paternal underweight group demonstrated a 3.462-fold higher risk for gross motor skill deficits. Similarly, in social–emotional development, SGA children in the paternal obesity group exhibited a 3.001-times greater risk of compromised social–emotional outcomes. No significant associations were observed between the paternal overweight and normal-weight groups across any developmental domains assessed by the ASQ-3 and ASQ: SE Questionnaires (Figure 2).

## 4. Discussion

This study grouped SGA infants by paternal pre-pregnancy BMI. Paternal underweight correlated with lower birth weight and severe SGA incidence. Offspring of obese fathers demonstrated higher BMI Z-scores and obesity risk versus normal-weight fathers’ children. Neurodevelopmental deficits in gross motor, communication, and social–emotional domains were observed in obese fathers’ offspring. Paternal underweight was linked to gross motor impairments, while paternal overweight showed no long-term growth/neurodevelopmental effects.

This study is the first to observe that while there was no significant difference in birth weight between SGA children of obese fathers and those of normal-weight fathers, the offspring of obese fathers exhibited significantly higher obesity rates and comprehensive neurodevelopmental delays. The increased obesity risk in SGA children may be attributed to obese fathers influencing their offspring’s gene expression and metabolism through altered epigenetic markers in sperm [34], such as DNA methylation and non-coding RNA expression, while also transmitting genetic predispositions to obesity [35]. These findings align with the results of Raad et al. [36] and Fullston et al. [37]. Concurrently, the intergenerational cycle of obesity driven by obesogenic dietary patterns in paternal obesity households may contribute to cumulative increases in obesity risk. While such effects remain latent during the fetal period, they gradually manifest as children develop autonomous eating behaviors, ultimately leading to heightened obesity susceptibility [38]. However, it remains unclear whether the obesity in these SGA children stems primarily from epigenetic factors or postnatal dietary habits. Future studies could incorporate clinical research on postnatal dietary patterns and experimental investigations to elucidate the underlying mechanisms. Notably, animal studies have revealed that offspring of obese male mice exhibit reduced expression of brain-derived neurotrophic factor mRNA in hippocampal samples—a gene critically involved in spatial learning and cognitive functions [39]. Conversely, paternal caloric restriction in rats significantly alleviated anxiety-like behaviors and socioemotional developmental deficits in first-generation adult offspring [40]. In our cohort, the paternal obesity group demonstrated the highest childhood obesity prevalence. Importantly, children with obesity are more prone to adverse psychological states (e.g., low self-esteem and body dissatisfaction), which may drive unhealthy dietary patterns and sedentary behaviors while simultaneously impairing gross motor skills [41]. This dual burden likely exacerbates weight gain, thereby creating a self-perpetuating cycle of obesity. This mechanistic interplay may elucidate our findings of heightened risks for gross motor impairment, problem-solving deficits, and socioemotional abnormalities in SGA children of obese fathers. This study is the first to systematically unveil the unique impact patterns of paternal obesity on neurodevelopment in SGA children, underscoring the critical role of paternal health status in optimizing eugenic practices and prenatal/postnatal care.

Additionally, this study revealed that paternal underweight was associated with the lowest offspring birth weights and an increased risk of severe SGA status, consistent with Li et al.’s findings demonstrating an independent paternal underweight–SGA association [42]. Mechanistically linked to paternal gut microbiota dysbiosis in humans, emerging evidence from animal models suggests that such disturbances can increase risks of low birth weight and severe growth restriction in offspring [43]. This provides experimental support for the potential biological plausibility of the paternal microbiome pathway implicated in our human findings. Furthermore, the observed gross motor development impairment in SGA children of the paternal underweight group may be linked to the elevated incidence of severe SGA within this cohort. Emerging evidence suggests that structural neurodevelopment—particularly cortical volume expansion and white matter integrity—is disproportionately vulnerable to the direct biological impacts of intrauterine growth restriction. This selective vulnerability appears to prioritize brain regions critical for motor coordination and social emotion processing, including the hippocampus, essential for sensorimotor integration, and the cerebellum, central to motor skill automation [44]. Importantly, however, SGA children born to underweight fathers exhibited no developmental disparities in communication, problem-solving, or socioemotional skills compared to those with normal-weight fathers. This may be attributed to the postnatal catch-up growth in physical development among SGA children of the paternal underweight group, resulting in body weight parity with their age-matched SGA peers from the paternal normal-weight group at follow-up [45,46]. Notably, studies report that approximately 90% of SGA infants achieve physical catch-up growth by age 2—a milestone strongly associated with improved neurodevelopmental trajectories [3]. Therefore, these findings suggest that early interventions promoting catch-up growth may play a critical role in optimizing neurodevelopmental outcomes in SGA children of paternal underweight.

Additionally, the findings of this study indicate no significant differences in birth weight or 24–36 months’ physical/neurodevelopmental outcomes between SGA children born to fathers with overweight and those born to fathers with normal weight. However, the 2–3 years follow-up period represents a critical limitation, as this duration may fail to capture long-term BMI-related epigenetic effects and neurodevelopmental trajectories, necessitating extended follow-up to clarify the dynamic impacts of paternal overweight on SGA offspring.

Historically, insufficient clinical emphasis on optimizing nutritional status across the reproductive continuum from parental preconception to offspring development has garnered significant policy attention. The National Academies of Science, Engineering, and Medicine’s 2020 expert workshop highlighted accumulating evidence on the interplay between maternal nutritional patterns, environmental exposures, and lactation dynamics, emphasizing their multigenerational health implications [47]. Concurrently, Soubry et al. proposed the groundbreaking “Paternal Origins of Health and Disease (POHaD)” concept [17], underscoring fathers’ critical role in transmitting environmental signatures to offspring through germline epigenetic mechanisms. Our methodologically robust study was the first to establish a U-shaped association (*p* < 0.05) between paternal preconception BMI extremes < 18.5 or ≥28 kg/m^2^ and suboptimal neurodevelopmental outcomes in SGA offspring. Based on these findings, we advocate for the following: (1) mandatory paternal BMI integration into standardized prenatal risk assessment protocols and (2) development of targeted early intervention strategies combining nutritional optimization, epigenetic monitoring, and developmental surveillance for families with paternal adiposity or undernutrition phenotypes.

## 5. Strengths and Limitations

This study’s strengths include innovatively examining paternal pre-pregnancy BMI’s long-term effects on SGA children, extending paternal health research beyond maternal-focused frameworks, and revealing a U-shaped association between paternal BMI extremes and suboptimal neurodevelopment in SGA offspring, addressing a critical intergenerational research gap. Limitations involve potential selection bias from a single-center design, reliance on parent-reported ASQ screening impacting developmental impairment measurement accuracy, and confounding from postnatal factors (e.g., parent–infant interactions and dietary patterns), environmental heterogeneity, and absent nutritional monitoring. Additionally, fathers’ BMI data, derived from self-reported weight and height obtained primarily through phone calls, may be subject to recall bias, as fathers were not consistently present during outpatient visits. This could affect the reliability of the measurements. Furthermore, the outcome assessment was conducted over a relatively wide postnatal age range (24 to 36 months). Although Z-scores were applied to mitigate the effects of variation, this approach may still compromise the robustness of the findings. Future studies should integrate tools like Bayley Scales and adopt multicenter cohorts with larger samples and extended follow-ups to strengthen generalizability and causality. In addition, to enhance the accuracy of paternal height and weight measurements and mitigate recall bias, future research should incorporate direct anthropometric assessments during clinical or study visits, using calibrated equipment, and ensure active father participation through incentives or scheduling flexibility.

## 6. Conclusions

This study of 412 SGA infants identified U-shaped associations (*p* < 0.05) between paternal preconception BMI extremes < 18.5 or ≥28 kg/m^2^ and offspring outcomes. Compared to paternal normal-weight status (BMI 18.5–24.9 kg/m^2^), paternal obesity elevated offspring obesity risk and multi-domain neurodevelopmental deficits, while paternal underweight correlated with lower birth weight, increased severe SGA incidence, and gross motor impairment risks. SGA children of normal-weight fathers had lower risks of obesity and neurodevelopmental issues. These findings establish paternal weight management as an intervention target for optimizing neurodevelopmental outcomes in SGA children. To translate these results into practice, we recommend the following: 1. Integrating paternal pre-pregnancy BMI screening into standardized prenatal risk assessment protocols alongside maternal assessments. Identifying fathers with underweight BMI < 18.5 kg/m^2^ or obesity BMI ≥ 28 kg/m^2^ should trigger enhanced monitoring and early intervention pathways for their offspring. 2. Developing targeted early intervention programs for families where the father has undernutrition or obesity. These programs should combine nutritional optimization for both parents’ preconception/prenatal health, developmental surveillance focusing on gross motor skills and socioemotional domains (especially for offspring of obese fathers), and exploration of epigenetic counseling where feasible.

## Figures and Tables

**Table 1:** Demographic characteristics of mothers stratified by paternal pre-pregnancy BMI.

	Paternal Pre-Pregnancy BMI Category					
Variable	Normal Weight (*n* = 188)	Underweight (*n* = 46)	Overweight (*n* = 124)	Obese (*n* = 54)	*H/χ*2	*p* Values
Paternal age at delivery, years	34.2 ± 4.5	33.8 ± 4.3	33.4 ± 4.2	32.6 ± 3.1	2.062	0.105
Maternal age at delivery, years	32.7 ± 3.8	33.4 ± 4.2	31.9 ± 3.9	32.7 ± 3.6	1.953	0.120
Maternal age group, *n* (%)					4.631	0.201
<35 years	137 (72.9)	27 (58.7)	90 (72.6)	35 (64.8)		
≥35 years	51 (27.1)	19 (41.3)	34 (27.4)	19 (35.2)		
Maternal BMI group, *n* (%)					12.214	0.202
Underweight	28 (14.9)	6 (13.0)	12 (9.7)	6 (11.1)		
Normal weight	104 (55.3)	20 (43.5)	70 (56.4)	22 (40.8)		
Overweight	40(21.3)	11 (23.9)	29 (23.4)	20 (37.0)		
Obese	16 (8.5)	9 (19.6)	13 (10.5)	6 (11.1)		
GWG group, *n* (%)					11.618	0.071
Inadequate GWG	46 (24.5)	9 (19.6)	29 (23.4)	14 (25.9)		
Adequate GWG	97 (51.6)	27 (58.7)	49 (39.5)	30 (55.6)		
Excessive GWG	45 (23.9)	10 (21.7)	46 (37.1)	10 (18.5)		
Bachelor’s degree or above						
Mother, *n* (%)	130 (69.1)	28 (60.9)	82 (66.1)	38 (70.4)	1.463	0.691
Father, *n* (%)	116 (61.7)	30 (65.2)	90 (72.6)	33 (65.3)	4.393	0.222
Primary caregiver, *n* (%)	77 (41.0)	18 (39.1)	56 (45.2)	21 (38.9)	0.953	0.813
Cesarean section, *n* (%)	50 (26.6)	13 (28.3)	40 (32.3)	15 (27.8)	1.202	0.753
Pregnancy with hypertension, *n* (%)	40 (21.3)	14 (30.4)	23 (18.5)	7 (13.0)	5.045	0.169
Pregnancy with preeclampsia, *n* (%)	40 (21.3)	11 (23.9)	29 (23.4)	9 (16.7)	1.167	0.761
Gestational diabetes mellitus, *n* (%)	30 (16.0)	8 (17.4)	35 (28.2)	11 (20.4)	7.221	0.065
Pregnancy with hypothyroidism, *n* (%)	28 (14.9)	9 (19.6)	31 (25.0)	10 (18.5)	4.991	0.172
Pregnancy with connective tissue disease, *n* (%)	7 (3.7)	5 (10.9)	6 (4.8)	3 (5.6)	3.273	0.351
Nuchal cord, *n* (%)	47 (25.0)	15 (32.6)	39 (31.5)	12 (22.2)	2.921	0.404
Oligohydramnios, *n* (%)	24 (12.8)	12 (26.1) ^d^	25 (20.2)	4 (7.4) ^b^	9.603	0.022 *
Abnormal placenta, *n* (%)	6 (3.2)	3 (6.5)	10 (8.1)	3 (5.6)	3.703	0.295

^b^: In comparison with underweight group, *p* < 0.05; ^d^: In comparison with obese group, *p* < 0.05. *: Four-group comparison, *p* < 0.05. Abbreviations: BMI, body mass index; GWG, gestational weight gain.

**Table 2:** Perinatal condition of SGA infants stratified by paternal pre-pregnancy BMI.

	Paternal Pre-Pregnancy BMI Category					
Variable	Normal Weight (*n* = 188)	Underweight (*n* = 46)	Overweight (*n* = 124)	Obese (*n* = 54)	*H/χ*2	*p* Values
Gestational age (week)	37.5 ± 2.4	37.1 ± 1.8	37.4 ± 2.4	37.3 ± 2.6	2.252	0.082
Preterm birth, *n* (%)	50 (26.6)	17 (37.0)	37 (29.8)	17 (31.5)	2.103	0.551
Birth weight (g)	2273 ± 492 ^b^	2077 ± 410 ^a,d^	2162 ± 515	2267 ± 503 ^b^	3.304	0.020 *
Length at birth (cm)	45 ± 4	44 ± 2	45 ± 4	45 ± 4	1.171	0.320
Male, *n* (%)	92 (48.9)	25 (54.3)	77 (62.1)	24 (44.4)	6.983	0.072
Severe SGA, *n* (%)	42 (23.6) ^b^	20 (43.5) ^a,c^	28 (22.6) ^b^	13 (24.1)	8.750	0.033 *
Neonatal asphyxia, *n* (%)	15 (8.0)	5 (10.9)	17 (13.7)	7 (13.0)	2.947	0.400

^a^: In comparison with normal-weight group, *p* < 0.05; ^b^: In comparison with underweight group, *p* < 0.05; ^c^: In comparison with overweight group, *p* < 0.05; ^d^: In comparison with obese group, *p* < 0.05. *: Four-group comparison, *p* < 0.05. Abbreviations: BMI, body mass index; SGA, small for gestational age.

**Table 3:** Comparison of long-term physical and neurological development of SGA offspring stratified by paternal pre-pregnancy BMI.

	Paternal Pre-Pregnancy BMI Category					
Variable	Normal Weight (*n* = 188)	Underweight (*n* = 46)	Overweight (*n* = 124)	Obese (*n* = 54)	*H/χ*2	*p* Values
Follow-up months	30.4 ± 3.9	29.4 ± 3.8	30.9 ± 4.3	30.9 ± 4.1	0.176	0.913
Weight Z-score	0.15 ± 0.67	0.22 ± 0.54	0.24 ± 0.76	0.42 ± 0.63	2.279	0.079
Height Z-score	0.14 ± 0.64	0.20 ± 0.61	0.15 ± 0.64	0.11 ± 0.60	0.187	0.905
BMI Z-score	−0.07 ± 1.02 ^d^	0.06 ± 0.99	0.21 ± 0.96	0.33 ± 1.01 ^a^	3.165	0.024
Emaciation, *n* (%)	20 (10.6)	4 (8.7)	8 (6.5)	3 (5.6)	2.436	0.487
Overweight, *n* (%)	15 (8.0)	3 (6.5)	18 (14.5)	6 (11.1)	4.164	0.244
Obesity, *n* (%)	2 (1.1) ^d^	1 (2.2)	4 (3.2)	5 (9.3) ^a^	8.068	0.045 *
ASQ-3 (Z-score)						
Communication	0.21 ± 0.46	0.27 ± 0.40	0.11 ± 0.38	0.20 ± 0.38	1.806	0.145
Gross motor	−0.34 ± 0.62 ^b,d^	−0.56 ± 0.59 ^a,c^	−0.33 ± 0.64 ^b,d^	−0.59 ± 0.53 ^a,c^	6.845	<0.001 *
Fine motor	0.08 ± 0.49	0.07 ± 0.53	0.12 ± 0.43	0.13 ± 0.34	0.317	0.813
Problem-solving	0.20 ± 0.41 ^d^	0.06 ± 0.42	0.06 ± 0.34	0.01 ± 0.40 ^a^	3.734	0.011 *
Personal–social	−0.10 ± 0.61	−0.04 ± 0.52	−0.12 ± 0.63	−0.19 ± 0.65	0.637	0.592

^a^: In comparison with normal-weight group, *p* < 0.05; ^b^: In comparison with underweight group, *p* < 0.05; ^c^: In comparison with overweight group, *p* < 0.05; ^d^: In comparison with obese group, *p* < 0.05. *: Four-group comparison, *p* < 0.05. Abbreviations: BMI, body mass index; ASQ-3, Ages and Stages Questionnaire, Third Edition.

## Data Availability

Availability of data and materials data are available upon reasonable request.
